# Development of Artificial Intelligence Systems for Chronic Kidney Disease

**DOI:** 10.31662/jmaj.2024-0090

**Published:** 2024-09-06

**Authors:** Eiichiro Kanda

**Affiliations:** 1 Department of Health Data Science, Kawasaki Medical School, Kukrashiki, Japan

**Keywords:** machine learning, prognosis, chronic kidney disease, dialysis, AI, natural language processing, category theory, guidelines

## Abstract

Chronic kidney disease (CKD) is a complex disease that is related not only to dialysis but also to the onset of cardiovascular disease and life prognosis. As renal function declines with age and depending on lifestyle, the number of patients with CKD is rapidly increasing in Japan. Accurate prognosis prediction for patients with CKD in clinical settings is important for selecting treatment methods and screening patients with high-risk. In recent years, big databases on CKD and dialysis have been constructed through the use of data science technology, and the pathology of CKD is being elucidated. Therefore, we developed an artificial intelligence (AI) system that can accurately predict the prognosis of CKD such as its progression, the timing of dialysis introduction, and death. Aiming for its social implementation, the prognosis prediction system developed for patients with CKD was released on the website. We then developed a clinical practice guideline creation support system called Doctor K as an AI system. When creating clinical practice guidelines, huge amounts of manpower and time are required to conduct a systematic review of thousands of papers. Therefore, we developed a natural language processing (NLP) AI system to significantly improve work efficiency. Doctor K was used in the preparation of the guidelines of the Japanese Society of Nephrology. Furthermore, by comparing and analyzing the medical word virtual space constructed by the NLP AI system based on patient big data, we proved using the latest mathematical theory (category theory) that this system reflects the pathology of CKD. This suggests the possibility that the NLP AI system can predict patient prognosis. We hope that, through these studies, the use of AI based on big data will lead to the development of new treatments and improvement in patient prognosis.

## Introduction

Chronic kidney disease (CKD) is a complex disease that is related not only to dialysis but also to the onset of cardiovascular disease and life prognosis. As renal function declines with age and depending on lifestyle, the number of patients with CKD is increasing worldwide. How to prevent the onset and suppress the progression of CKD is an important issue from the perspective of patients and medical costs. In recent years, artificial intelligence (AI) and information and communication technology (ICT) have developed, and these technologies are being incorporated into the studies of the pathology and treatment of CKD.

If patients’ prognosis can be accurately predicted, those with a poor prognosis can be detected, and medical resources such as testing and treatment can be focused on such patients. In addition, since it is possible to predict changes in prognosis depending on the choice of treatment method, it becomes possible to select the best treatment method, which can be used as a reference when considering drug combinations and deciding when to introduce dialysis. Therefore, in this paper, we will mainly outline the research studies on CKD that we have conducted using AI and ICT based on patient databases.

## Promotion of AI and ICT Research through Academic Societies

Academic societies in Japan have been carrying out various activities related to AI and ICT. The Japanese Society of Nephrology (JSN) has established an AI and ICT Utilization Infrastructure Subcommittee that aims to promote the use of AI and ICT in order to overcome kidney diseases ^(1)^. With the recent development of database technology, analyzing big data has become possible, which was previously impossible. JSN has constructed Japan-CKD Database, which collects data on patients with CKD from university hospitals across the country ^(2)^.

There are approximately 350,000 dialysis patients nationwide, and it is important to collect information on their characteristics. The Japanese Society for Dialysis Therapy (JSDT) has been conducting a statistical survey [JSDT Renal Data Registry (JRDR)] since 1966, and it has been expanding its database based on this survey ^(3)^.

Moreover, the Japanese Society for Artificial Intelligence in Nephrology and Blood Purification was established and has been promoting research using AI.

## Predicting Progression to Early CKD in Health Checkup Participants

Various prognostic indicators for CKD and dialysis patients have been reported; however, there have only been a few reports of machine learning models that use patient big data to predict the prognosis of CKD patients. Therefore, we used a database related to CKD to create a model that predicts the prognosis of CKD and dialysis patients.

Unlike in chronic glomerulonephritis and diabetic nephropathy, proteinuria is often not detectable in nephrosclerosis, thus making its early detection difficult. Many patients with such potential risks are left untreated for several years because initial medical examinations do not show a decrease in glomerular filtration rate and they often seek medical attention after their serum creatinine levels rise. Therefore, we conducted a study to predict the risk of developing CKD using machine learning on health checkup data ^(4)^.

**Figure 1.**
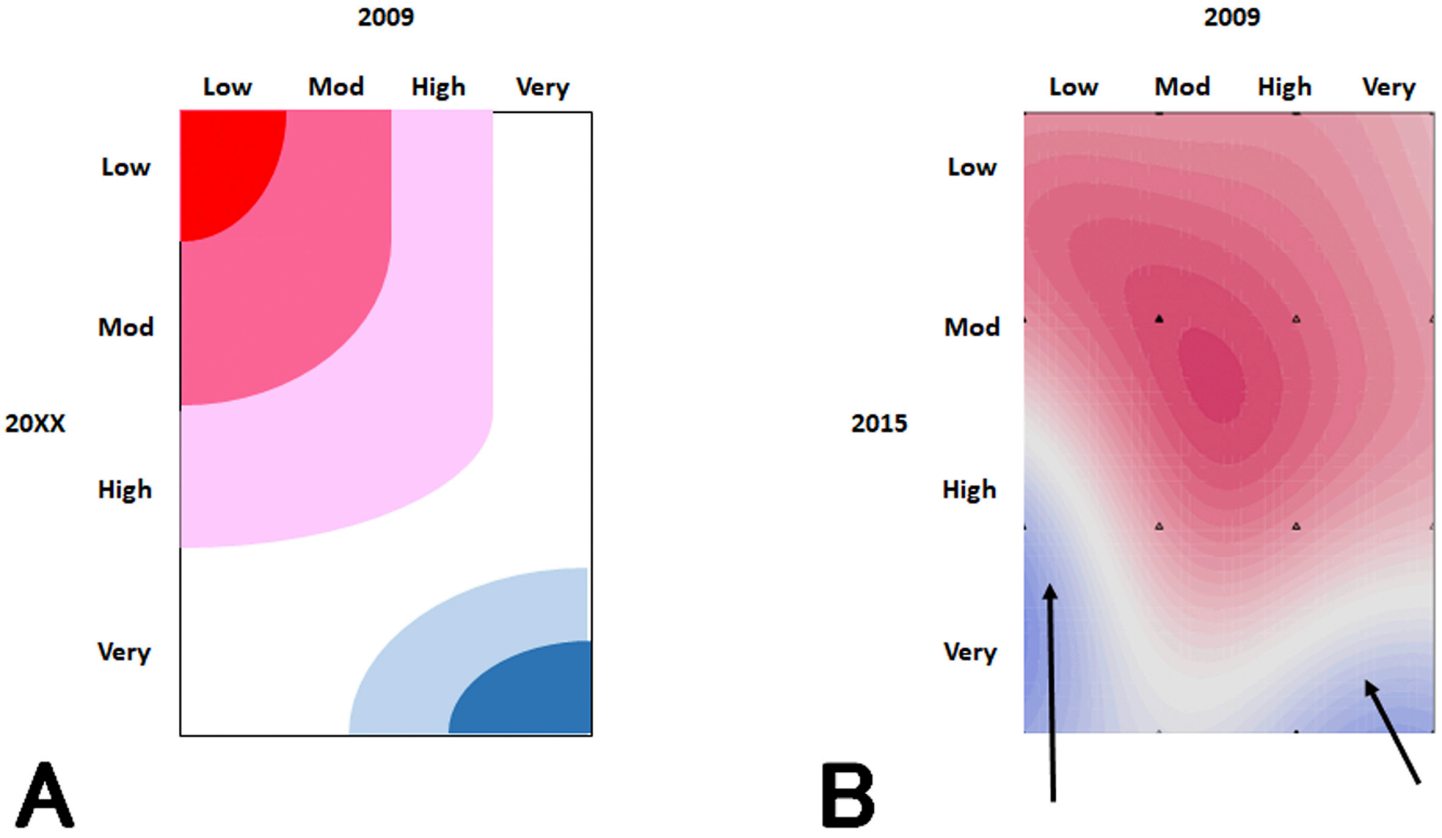
Predicted probability distribution of CKD progression determined from changes in CKD severity classification. The heat map shows the patient distribution according to CKD severity classification predicted by SVM, with blue representing high-probability distribution and red representing low-probability distribution. A. Patient distribution prediction: The horizontal axis represents the 2009 CKD severity classification, and the vertical axis represents the expected CKD severity classification in 20xx. B. Patient distributions in 2009 and 2015: The horizontal axis represents the 2009 CKD severity classification, and the vertical axis represents the 2015 CKD severity classification. Even if a patient was at low risk in 2009, they may become at high risk later (→ in Figure B).

From the obtained database, various background factors such as blood pressure, weight, and the presence or absence of diabetes were intricately involved in the decline of renal function. In addition, the outcome incidence rate was very low, so conventional statistical analyses could not be used. Therefore, instead of analyzing using conventional frequentist statistics, we used a Bayesian network to search for risk factors, trained a support vector machine (SVM), and then displayed the distribution of the patient’s CKD progression probability in a heat map (Figure 1).

It was expected that the probability of CKD progression in patients whose risks were low at the start of the study would remain low for years (Figure 1A), but the SVM revealed that the number of high-risk patients gradually increased (Figure 1B). This finding shows that even if a patient is at low risk after a single physical examination, it is not guaranteed that they remain so for years to come; therefore, it is necessary to have a follow-up observation for 3 years or more. Even in research studies like this, in which analyses using general statistics are difficult, machine learning makes it possible to analyze big data.

## Prediction of Prognosis of Dialysis Patients

The state of protein-energy wasting (PEW) is one of the characteristics of malnutrition in dialysis patients. In PEW, the combination of malnutrition and inflammation has a negative impact on life prognosis. Therefore, we developed a machine learning model to detect high-risk patients using JRDR ^(5)^.

**Figure 2.**
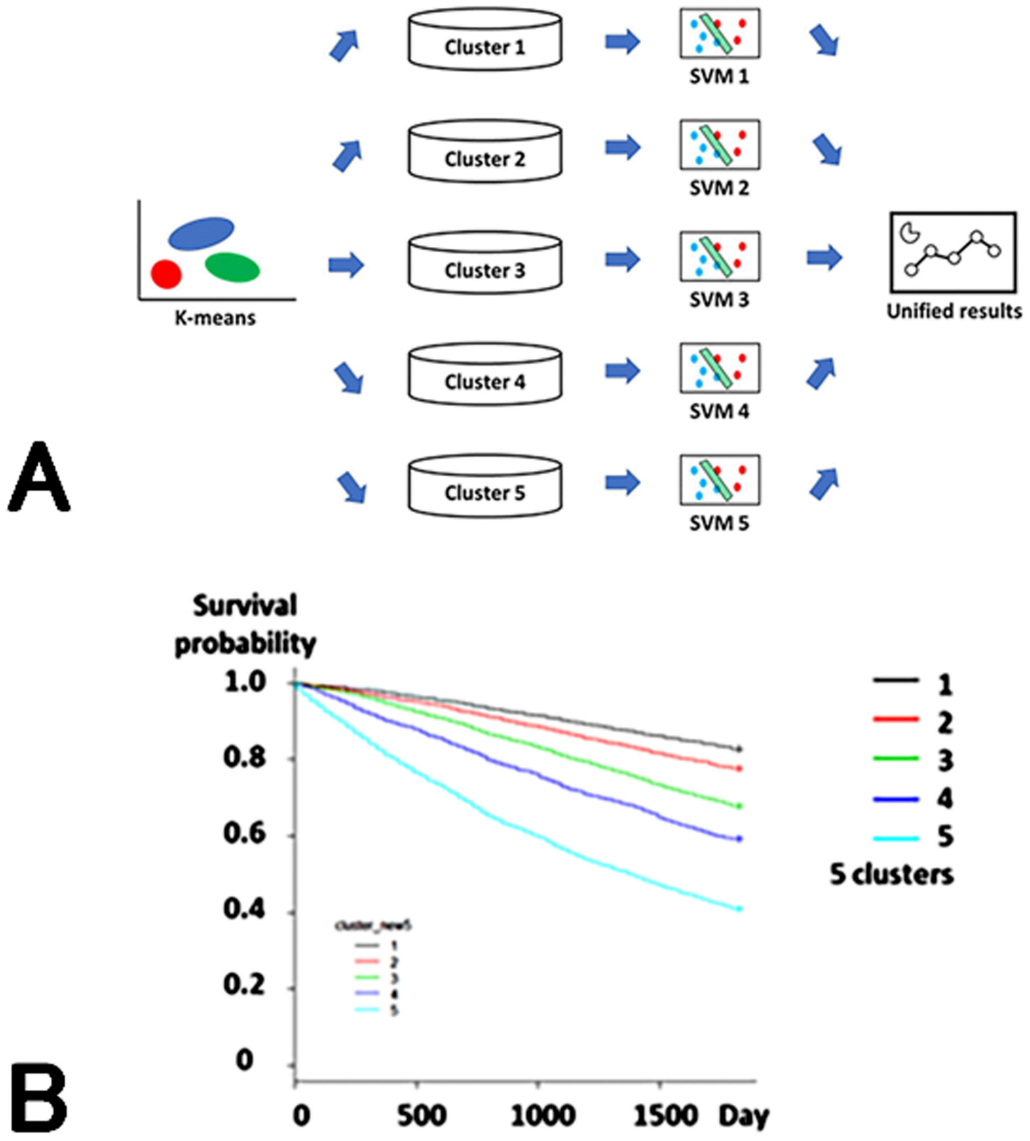
Ensemble model for prediction of prognoses of dialysis patients. A. Model structure: The ensemble model created in this research study consists of five groups and an SVM for each. Patients are classified into one group using the k-means method and analyzed using SVM. Prognosis is predicted from the integrated results. B. Relationship between cluster and 5-year survival rate: Group 1 had the best prognosis and Group 5 had the worst prognosis.

Machine learning models have extremely high accuracy in classifying and predicting data. However, unlike traditional statistical models, the analysis results are often difficult for humans to understand (black box). Therefore, we created an ensemble model consisting of a combination of multiple machine learning models to make the analysis content understandable to some extent. In this model, patients were classified into five groups using the k-means method, and then the 1-year mortality for each group was predicted using SVM and the results were integrated (Figure 2A).

Group 1 consisted of young men with glomerulonephritis as their primary disease, Group 2 consisted of women with glomerulonephritis, Group 3 consisted of patients with diabetic nephropathy, Group 4 consisted of elderly patients with nephrosclerosis, and Group 5 consisted of elderly patients with PEW.

When the relationship between a 1-year mortality risk and group was examined, a trend toward a worse life prognosis was observed in the order of Groups 15, with the hazard ratio for Group 5 being 8.86 (95% CI 7.68, 10.21) (Figure 2B). The prediction accuracy [area under receiver operator characteristic curve (AUROC)] was 0.948, which was higher than those of logistic regression models and deep learning.

In this study, we found that machine learning automatically classified patients in line with previous evidence and the clusters reflected prognosis. This suggests that machine learning models can learn in accordance with the evidence that humans have obtained so far.

## Prediction of Complications of Hyperkalemia

Renin-angiotensin-aldosterone system (RAAS) inhibitors are often administered to patients with CKD not only to lower blood pressure but also to slow the progression of CKD and reduce urinary protein levels. Hyperkalemia frequently occurs in patients with CKD owing to the effects of RAAS inhibitors and decreased renal function. With the onset of hyperkalemia, RAAS inhibitors are often discontinued, which can lead to a decreased renal function and an increased risk of developing cardiovascular disease ^(6)^. In clinical practice, patients are often unsure of whether to continue or discontinue RAAS inhibitors as a treatment option. In such cases, being able to predict the onset of complications associated with hyperkalemia, such as cardiovascular disease, would be useful in determining treatment options.

We used data from hyperkalemic patients (n = 24,949) to predict mortality, the timing of introduction of renal replacement therapy (RRT), hospitalization for heart failure (HHF), and cardiovascular events within 3 years of a hyperkalemic episode. We created a model to predict the above using ^(7)^. The AUROCs of the created model were 0.823, 0.957, 0.863, and 0.809 for death, RRT, HHF, and cardiovascular events, respectively, which showed higher accuracy than the logistic regression model.

In addition, CKD is a risk factor for death in diabetic patients. Preventing its onset is an important therapeutic target. Therefore, we created a model ^(8)^. The accuracy of predicting the onset of CKD or heart failure was 0.718, and the patient group predicted to be at high risk had a statistically significant higher event incidence rate (*p* < 0.0001). Furthermore, SHapley Additive exPlanations analysis revealed that frequent outpatient visits, age, frequent hospitalizations, and use of diuretics were risk factors. Machine learning models can rank variables that contribute to outcome prediction, making them useful for searching for risk factors when statistical models cannot be applied.

## Practical Application of Prognosis Prediction System for Patients with CKD

Machine learning models have been developed to predict the progression of CKD, but none have been put into practical use. Therefore, we developed a model to predict the probability of dialysis initiation or death in patients with CKD patients and attempted to put it into clinical use ^(9)^.

In this study, we used cohort study data (n = 3,714) of CKD patients to create 16 models for predicting dialysis initiation or death and compared their prediction accuracies. In addition, considering the user’s effort, we used patient background and drugs that are easily available at outpatient clinics as input and selected variables on the basis on their importance in each model.

**Figure 3.**
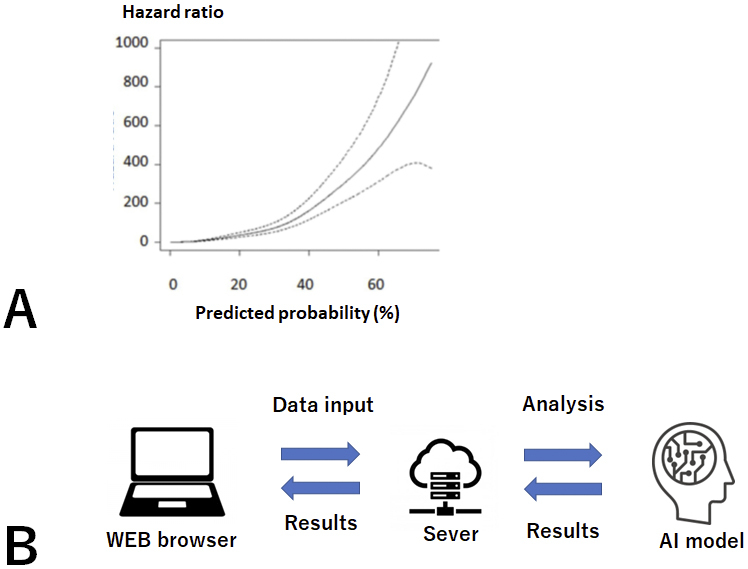
Mechanism of prognosis prediction system. A. Relationship between probability predicted by machine learning model and risk of outcome occurrence: A Cox proportional hazards model adjusted for patient characteristics was used. The predicted probability of the RF_all model correlated well with the risk of occurrence of the outcome (* p* < 0.0001). B. System configuration: The input data is analyzed by a machine learning model via the server, and the results are displayed.

Random forest (RF) models using time-series data sets, the model using all variables (RF_all), and the model selecting eight variables (RF_v8) showed a high accuracy and a strong correlation with the occurrence of actual outcomes (*p* < 0.0001) (Figure 3A). In addition, given that these models will be used for CKD patients with various underlying diseases and background factors, we compared the predictive ability of the models in patient groups, such as those in CKD stages G4-G5, those with diabetes, and elderly patients. Here, RF_all and RF_v8 also showed the highest accuracy.

We then developed and put into practical use a Web-based prognosis prediction system. This system calculates risk on the basis of data entered by the user, and it shows the predicted risk and a message in a Web browser (Figure 3B). As of 2024, the system is publicly available (URL http://133.242.150.48:8000/).

In clinical practice, treatment plans are selected while taking trade-offs into consideration. For example, should a low-protein diet be prescribed to prevent the progression of hyperphosphatemia and renal damage, or should a high-protein diet be prescribed to prevent malnutrition/sarcopenia/frailty? It may be difficult to make such a decision. Probabilities estimated by machine learning models are useful in these situations. A prognosis prediction system is considered helpful in quickly predicting prognosis and determining treatment strategies in outpatient settings.

Medical staff members are busy and need a system that is easily accessible and displays results quickly. However, such a system may not work owing to differences in OS among manufacturers. Our system solves these problems by limiting the input variables to those used in daily life and using a Web system.

Furthermore, this study revealed that there are various hurdles that should be overcome before prognosis prediction system can be put into practical use, such as data collection, development of a machine learning model, construction of a user-friendly system, and publication.

## Development and Use of CKD Guideline Creation Support System

How to suppress the progression and prevent the onset of CKD is an important issue from the perspective of patients and medical costs. The JSN has compiled CKD guidelines based on a collection of evidence regarding the pathology and treatment of CKD.

Regarding the evidence that has been collected so far in relation to CKD, many basic and clinical studies have been conducted both in Japan and overseas, and a vast amount of knowledge has been accumulated and reported. According to PubMed, there were approximately 10,000 literature searches for “chronic kidney disease” in 2023 alone. When creating guidelines, the literature must be comprehensively evaluated and adopted by doctors. However, it is nearly impossible for doctors in charge to search for evidence on CKD from the entire domestic and international medical literature. Therefore, we developed a system that supports guideline creation using natural language processing (NLP) AI technology.

## Procedure for Creating Guidelines

In medical guidelines, after collecting as much evidence (medical papers) related to a clinical question (CQ) as possible and conducting a systematic review (SR), statements are compiled on the basis of results ^(10)^.

The SR procedure begins with a primary screening, in which a bibliography list is created that includes a wide range of papers related to CQ from databases such as MEDLINE, and papers related to CQ are selected based on their abstracts. As a secondary screening, the selected papers are then read and those that meet the criteria are selected. The reviewers then evaluate the treatment effects and bias of each selected article and evaluate the overall evidence.

Until now, SR has mainly been performed manually by humans. However, in such a comprehensive literature search, the number of papers listed can be extremely large, and the number of candidate papers may be in the thousands. Manually, selecting papers related to CQ from this list of papers based on their titles and abstracts takes effort and time, and it is also likely that some papers will be missed. Several systems have been reported to assist SR, but they are difficult to use because they have not been customized for Japanese guideline creation ^(11), (12), (13), (14), (15), (16)^. Therefore, we developed an AI-equipped analysis system (Doctor K) that aims to automatically create guidelines.

## Overview of Guideline Creation Support System

If we consider a collection of words to be a sentence, and a collection of sentences to be a document, then a paper can be considered a multidimensional array of word vectors *w*_i_. As a simple model, let us consider two papers as *D*_1_ = (*w*_1_^1,^ *w*_2_^1,^ …, *w*_m_^1^) and *D*_2_ = (*w*_1_^2^, *w*_2_^2^, …, *w*_n_^2^) . We can think of the document similarity as follows:


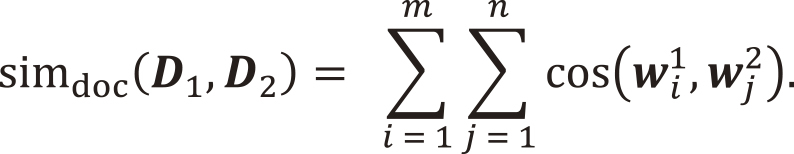


**Figure 4.**
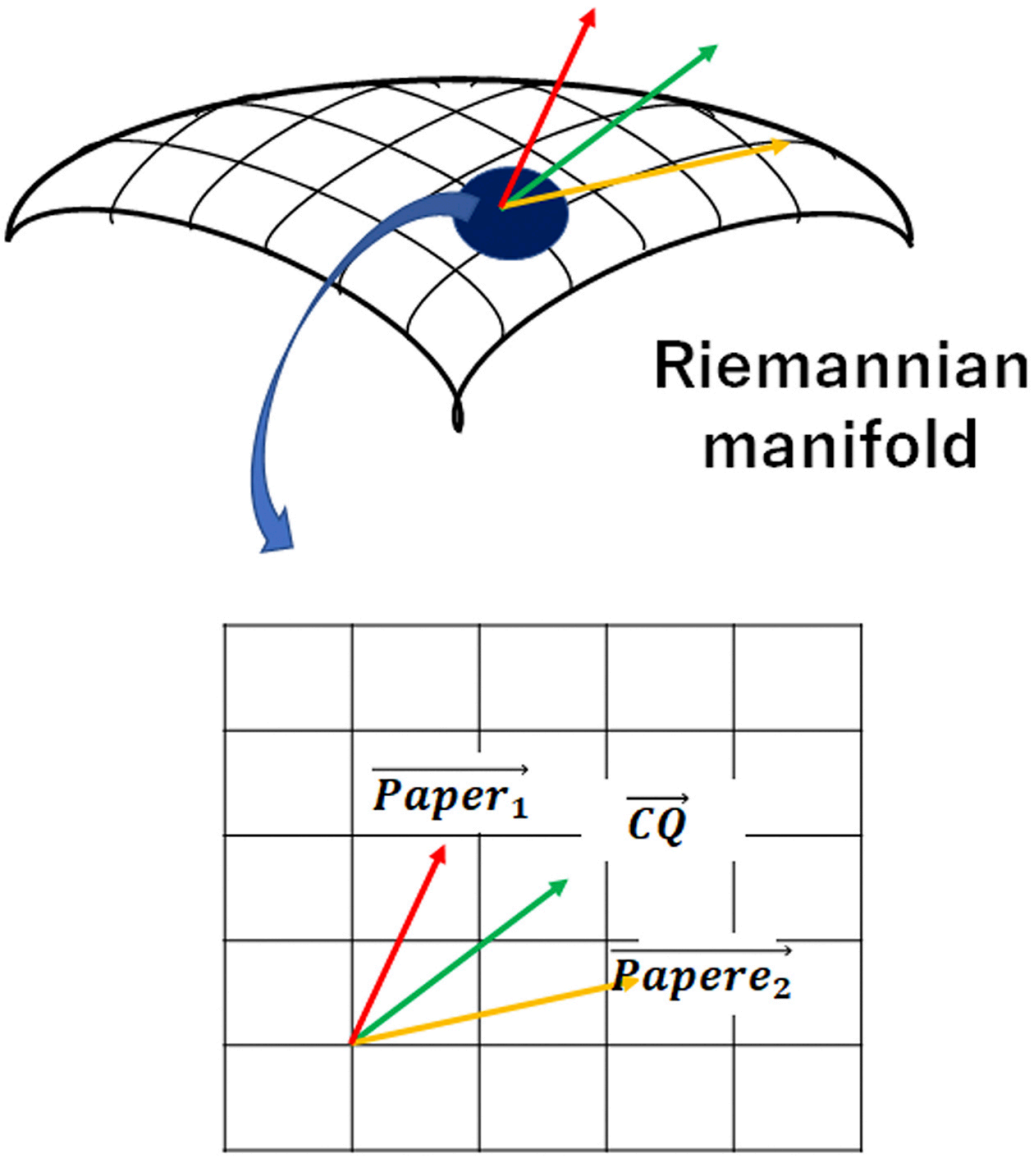
Structure of Doctor K. Doctor K’s AI analyzes papers and builds a Riemannian manifold. Since this Riemannian manifold is locally located in Euclidean space, it is possible to measure the distance between the paper and the CQ. CQ, clinical question.

In reality, it cannot be said with certainty that papers are distributed in a Euclidean space where the basis vectors are perpendicular to each other, and the relationships among documents must be evaluated assuming a non-Euclidean space. Therefore, to improve the accuracy of machine learning, it is necessary to evaluate the relationship between CQs and papers with reference to a Riemannian manifold, taking into account that the distance is determined in space when an inner product is applied to a tangent space (Figure 4).

By taking these points into account, we developed Doctor K, which can automatically select papers close to the CQ from multiple documents obtained from databases such as MEDLINE. After reading the text data, this system uses deep learning to instantly extract vectors of papers from distributed representations of words.

Doctor K was examined for CQs in the 2018 CKD Guidelines of the Japanese Society of Nephrology. A manual keyword search returned 1,012 papers, which were selected after several weeks of peer review by multiple reviewers. When this data was used as the correct answer data to train the system, Doctor K’s analysis results showed an accuracy rate of over 98%. This analysis showed papers that had been overlooked, demonstrating a significant reduction in the possibility of incorrect selection.

Previously, reviewers had to simultaneously work on Microsoft Excel and PubMed, which was inefficient. Therefore, we aimed to create a user-friendly screen layout. Furthermore, by learning users’ selection trends and making it possible to rank results in order of relevance to the CQ, the work time was significantly reduced compared with that in conventional SRs. Doctor K was used in ^(17), (18)^.

Through this development, it has been suggested that it may be possible to integrate evidence regarding CKD by comprehensively analyzing vast amounts of medical text data by text mining. In the future, we aim to develop a fully automatic SR system.

## Development of NLP AI System to Predict the Progression of CKD

Research proceeds on the premise that the study is scientifically correct. In addition, the relationships among medical words naturally reflect medical concepts and are considered to be building blocks of a network. However, the accuracy of the network of medical words is conceptual, and no research has examined how accurate it is or whether it matches actual patient data.

In recent years, the development of NLP AI system such as ChatGPT and Bard has been progressing and has become a hot topic. The current popularity of NLP began when a model called Word2Vec was shown not only to calculate words as vectors but also to preserve the meaning of words. NLP has been an effective technology for text mining to extract medical concepts and has been used in medical research studies ^(19)^. Several studies have shown the relationship of the appearance of medical words such as symptoms and medicines in electronic medical records with the risk of end-stage kidney disease (ESKD) ^(20)^. Recently, various types of NLP AI system have been developed, such as generative pretrained transformer 4 (GPT-4), which is used in ChatGPT ^(21)^.

Word2Vec is an early type of NLP AI system and is composed of neural networks for learning word associations from a large corpus of text. This is an efficient tool for obtaining high-quality distributed representations that embed words into vectors ^(22)^. This vectorization enables the calculation of word vectors and the acquisition of meaningful results. It has been reported that semantic information represents objects ^(23), (24)^. These lines of evidence suggest that medical-word vectors are expected to compose a network and are associated with real data of CKD patients and the pathophysiological concepts of CKD.

**Figure 5.**
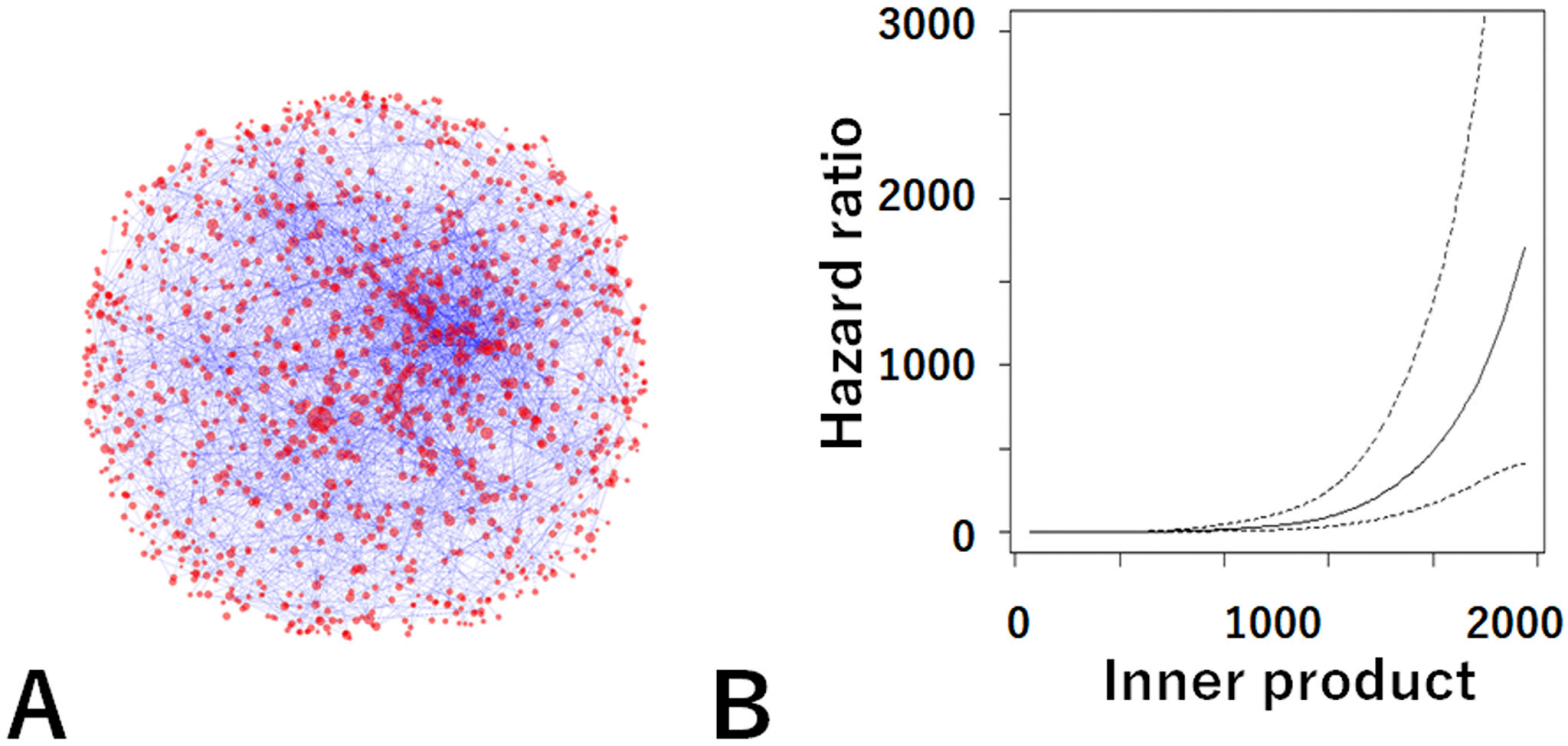
Medical word virtual space. A. Network of medical words in a medical-word virtual space. Red dots represent medical words, and the importance of a medical word is indicated by the size of the dot. Relationships among medical words are indicated by blue lines. B. The relationship (inner product) between the patient vector and the ESKD vector is strongly correlated with the risk of ESKD (*p* < 0.0001).

Therefore, we used Word2Vec to analyze 165,271 medical papers and built a network of medical words on a medical-word virtual space (Figure 5A) ^(25)^. To use an NLP AI system in clinical practice, it is necessary to ensure that the analysis behind it reflects medical knowledge. Therefore, we manually checked the relationships among medical words in the network and found that the analysis by the AI system ensured medical significance and vector calculation. This work was so laborious and difficult that no one had ever done it before.

We then confirmed the relationship between this network and actual CKD patient data (n = 26,433) using an analysis method based on the latest mathematical category theory. Category theory is used in the analysis of relationships among things and is attracting attention in data science and cognitive science. Our study was the world’s first attempt in the field of clinical research. This showed that the medical concepts in the paper matched actual patient data with high accuracy and that the medical-word network reflected the pathology of CKD. This study showed that the NLP AI system accurately reflects the actual disease pathology and its analysis is reliable.

In addition, the relationship (inner product) between the patient vector and the ESKD vector in the virtual medical-word space was discovered as a new prognostic indicator (Figure 5B). This established a fundamental theory for predicting the probability of future dialysis by inputting information such as elderly, gender, diabetes, use of antihypertensive drugs, and presence of urinary protein.

## Conclusion

The development of medical systems that use medical databases and machine learning is rapidly advancing. It is expected that the latest approaches using AI and ICT will lead to the development of new treatments that can be used in medical care and the improvement in the prognosis of CKD patients.

## Article Information

### Conflicts of Interest

None

### Sources of Funding

This research was supported by AMED under grant number JP24rea522003 and the Japan Society for the Promotion of Science (KAKENHI grant number JP 22K08346).

### Approval by Institutional Review Board (IRB)

This paper is a review and patient data was not used in it. Thus, there is no need of ethical approval.
